# Combining different bacteria in vaccine formulations enhances the chance for antiviral cross-reactive immunity: a detailed *in silico* analysis for influenza A virus

**DOI:** 10.3389/fimmu.2023.1235053

**Published:** 2023-08-22

**Authors:** Andrés Bodas-Pinedo, Esther M. Lafuente, Hector F. Pelaez-Prestel, Alvaro Ras-Carmona, Jose L. Subiza, Pedro A. Reche

**Affiliations:** ^1^Children’s Digestive Unit, Institute for Children and Adolescents, Hospital Clinico San Carlos, Madrid, Spain; ^2^Department of Immunology & O2, Faculty of Medicine, University Complutense of Madrid, Ciudad Universitaria, Pza. Ramón y Cajal, Madrid, Spain; ^3^Inmunotek, Alcalá de Henares, Spain

**Keywords:** MV130, bacteria, respiratory viruses, cross-reactivity, epitope, influenza A virus

## Abstract

Bacteria are well known to provide heterologous immunity against viral infections through various mechanisms including the induction of innate trained immunity and adaptive cross-reactive immunity. Cross-reactive immunity from bacteria to viruses is responsible for long-term protection and yet its role has been downplayed due the difficulty of determining antigen-specific responses. Here, we carried out a systematic evaluation of the potential cross-reactive immunity from selected bacteria known to induce heterologous immunity against various viruses causing recurrent respiratory infections. The bacteria selected in this work were Bacillus Calmette Guerin and those included in the poly-bacterial preparation MV130: *Streptococcus pneumoniae*, *Staphylococcus aureus*, *Staphylococcus epidermidis*, *Klebsiella pneumoniae*, *Branhamella catarrhalis* and *Haemophilus influenzae*. The virus included influenza A and B viruses, human rhinovirus A, B and C, respiratory syncytial virus A and B and severe acute respiratory syndrome coronavirus 2 (SARS-CoV-2). Through BLAST searches, we first identified the shared peptidome space (identity ≥ 80%, in at least 8 residues) between bacteria and viruses, and subsequently predicted T and B cell epitopes within shared peptides. Interestingly, the potential epitope spaces shared between bacteria in MV130 and viruses are non-overlapping. Hence, combining diverse bacteria can enhance cross-reactive immunity. We next analyzed in detail the cross-reactive T and B cell epitopes between MV130 and influenza A virus. We found that MV130 contains numerous cross-reactive T cell epitopes with high population protection coverage and potentially neutralizing B cell epitopes recognizing hemagglutinin and matrix protein 2. These results contribute to explain the immune enhancing properties of MV130 observed in the clinic against respiratory viral infections.

## 1 Introduction

Recurrent respiratory tract infections (RRTIs) are a leading cause of morbidity and mortality in children and adults (1, 2). The most common cause of RRTIs are seasonal respiratory viruses like human rhinovirus (HRV), respiratory syncytial virus (RSV) and influenza A and B viruses (IAV and IBV), among others (3). Management of these infections is challenging and the search for new preventive and therapeutic interventions is a subject of intense research (2, 4). In the absence of effective specific vaccines, an interesting strategy is the use of poly-bacterial preparations that can stimulate mucosal immunity and increase the host resistance to respiratory viral infections (5–7). A relevant example is MV130 that contains different species of inactivated whole-cell Gram-positive and negative bacteria (8). MV130 has been shown effective in reducing the number of RRTIs in both children and adults, including those of viral etiology (5, 9–11). Moreover, it has been shown that MV130 immunization protects mice from respiratory infections caused by influenza A (12) and severe acute respiratory syndrome coronavirus 2 (SARS-CoV-2) (13). Overall, these data indicate that MV130 can induce heterologous immunity against common respiratory viruses. The mechanism by which MV130 mediates protective heterologous immunity has been linked to the induction of trained immunity, much like with other bacteria-based formulations like Bacille Calmette-Guérin (BCG) vaccine (14–18). Trained immunity was originally defined as a kind of non-specific immunological memory acquired by innate immune cells, involving epigenetic and metabolic cell reprogramming. Trained immunity memory is short-lived, usually lasting less than 1 year, and is independent of T and B cells (19, 20). Although unexplored, MV130 could also be providing protection against respiratory viruses by inducing cross-reactive adaptive immunity.

Cross-reactive immunity occurs when preexisting memory T and B cells elicited by a particular antigen/infectious agent recognize and respond against different antigens/infections (21, 22). The occurrence of cross-reactive immunity between unrelated pathogens, including between bacteria and viruses, is well documented (21, 23–26) and it is facilitated by the poly-specificity of B and T cell receptors (27–30) and also by the recognition on small portions within the antigens (epitopes) (31). Cross-reactive immunity between MV130 and respiratory viruses remain yet to be verified, as it requires of precision immune monitoring, testing responses to all potential cross-reactive antigens and epitopes (32). However, the chance for cross-reactive immunity can be assessed *in silico*. To that end, in this work, we followed an approach consisting on identifying highly similar peptide sequences between antigen sources (identity ≥ 80%, over at least 8 residues) and subsequently predicting T or B cell reactivity (33, 34).

In this way, we obtained the shared peptidome between common respiratory viruses (IAV, IBV, HRV A, B and C, RSV A and B and SARS-CoV-2) and BCG and bacteria included in the MV130 formulation: *S. pneumoniae, S. aureus, S. epidermidis, K. pneumoniae, B. catarrhalis* and *H. influenzae*. Interestingly, the peptidome space that is shared between the specific bacteria in MV130 and viruses is non-overlapping, highlighting that combining bacteria in vaccine formulations enhance the chance for cross-reactive immunity. Given that MV130 heterologous immunity to influenza A virus (IAV) has been confirmed in mice models, we also determined the potential cross-reactive T and B cell epitopes with this virus. We found that MV130 contain numerous potentially cross-reactive T cell epitopes with high population protection coverage and accessible B cell epitopes in the virion membrane. Overall, these results support the hypothesis that MV130 could induce protective cross-reactive immunity against respiratory viruses, particularly to IAV.

## 2 Methods

### 2.1 Microbial proteomes

The entire proteomes of 7 bacteria species and 8 respiratory viruses were obtained from NCBI after the entries indicated in **Table 1** and assembled into individual files in FASTA format.

**Table 1:** Amino acid sequences from pathogens and vaccines considered in this study.

Pathogen	NCBI Accession	Proteins/CDS
Influenza A virus (IAV)	GCF_000865725	12
Influenza B virus (IBV)	GCF_000820495	10
Human rhinovirus A (HRVA)	NC_038311	1
Human rhinovirus B (HRVB)	NC_038312	1
Human rhinovirus C (HRVC)	NC_009996	1
Respiratory syncytial virus A (RSVA)	NC_038235	11
Respiratory syncytial virus A (RSVB)	NC_001781	11
Severe acute respiratory syndrome coronavirus 2 (SARS-CoV-2)	NC_045512	12
Bacille Calmette-Guérin (BCG)	GCF_000009445	4034
*Branhamella catarrhalis* (BCA)	GCF_000092265	1607
*Haemophilus influenzae* (HIN)	GCF_000027305	1597
*Klebsiella pneumoniae* (KPN)	GCF_000240185	5779
*Staphylococcus aureus* (SAU)	GCF_000013425	2767
*Staphylococcus epidermidis* (SEP)	GCF_000007645	2282
*Streptococcus pneumoniae* (SPN)	GCF_000007045	1861
MV130*		15893

* MV130 includes all bacteria but BCG.

### 2.2 Identification of shared peptides between microbial proteomes

To identify shared peptides between virus and bacteria proteomes, the entire viral proteomes were first fragmented into overlapping 17-mer peptides with a 10-residue overlap. Subsequently, the peptides were used as queries in sequence similarity searches using BLASTP (35) against the target bacteria proteomes, previously formatted as BLAST databases. BLAST searches were performed with default parameters and the *e*-value set to 10,000. BLAST results were processed and shared peptides were identified from ungapped hit alignments including 8 or more residues with ≥ 80% identity. BLAST searches and processing of BLAST results were performed using an *ad-hoc* PERL script that will be provided by Dr. Reche upon writing request.

### 2.3 Prediction of T and B cell epitopes

Peptides were assessed as potential T cell epitopes by predicting their binding to major histocompatibility complex (MHC) molecules. Peptide binding to MHC class I (MHC I) molecules was predicted using standalone versions of RANKPEP (36, 37) and NetMHCpan (38, 39). RANKPEP and NetMHCpan prediction models were selected to match the size of the peptides if they include 8 or 9 residues. The binding of peptides with more than 9 residues to MHC I molecules was assessed by evaluating that of all nested 9mer peptides. Binding of a peptide to a given MHC I molecule was considered to occur at a 2% Rank cutoff given by both RANKPEP and NetMHCpan, which allows selecting weak and strong binders % Ranks of test peptides are obtained by ranking their predicted binding affinity or binding scores compared to a large set of random peptides. The use of binding thresholds based on % Rank removes bias of certain molecules towards higher or lower predicted affinities and facilitates comparing and combining predictions by distinct methods. Binding of peptides to MHC class II (MHC II) was predicted using NetMHCIIpan (40) using a 10% Rank cutoff, which allows detecting strong and weak binding peptides. Human MHC II molecules targeted for predictions included HLA-DR, HLA-DQ, and HLA-DP molecules.

B cell epitopes were predicted using an standalone version of BepiPred1.0 (41). BepiPred reports antigenicity values per residue (*a_i_*), and a global B cell epitope score (*B*) was computed as indicated elsewhere (34, 42) consisting of the average *a_i_* value. Peptides with *B* value ≥ 0.4 were considered as antigenic or potential B cell epitopes.

### 2.4 Statistical analyses

χ^2^ tests were used as reported previously (43) to assess whether the distribution of the cross-reactive epitope sequences in proteins is proportional to the size of the proteins. The χ^2^-statistics value was computed using equation 1.


Eq. 1 $$\chi^{2}=\sum_{i=1}^{k}\frac{{\left(O_{i}-E_{i}\right)}^{2}}{E_{i}}\;$$

In this equation, *k* is the number of protein antigens, *O_i_* the number of observed epitopes in antigen *i*, and *E_i_* the number of expected epitopes in antigen *i* if they were distributed proportionally to the size of the proteins. The null, *H_0_*, hypothesis considers that epitopes are distributed proportionally to the size of proteins and it is rejected when the computed χ^2^-statistic value is above the χ^2^–distribution value at *k – 1* degrees of freedom and a given *α* value.

### 2.5 Other procedures

The percentage of the world population that could respond to CD8 and CD4 T cell epitopes (population coverage) was computed after their MHC binding profiles using a command line version of EPISOPT (44) and the IEDB PPC tool at http://tools.iedb.org/tools/population/iedb_input (45), respectively, considering the relevant allele expression for the entire world population. The presence of a C-terminus in potential CD8 T cell epitopes compatible with cleavage by the proteasome was predicted from the relevant antigens using PCPS at http://imed.med.ucm.es/Tools/pcps/ with default settings (46, 47). Ectodomains of hemagglutinin (HA), neuraminidase (NA) and matrix protein 2 (M2) from IAV (A/Puerto Rico/8) were identified from UNIPROT accession H2KIW3_9INFA, H2KIW6_9INFA and H2KIW4_9INFA, respectively. PyMOL Molecular Graphics System Version 2.4.1 Schrödinger, LLC was used to map peptide sequences in tertiary structure of influenza A virus hemagglutinin (HA) (PDB: 1RU7) and to generate molecular renderings. Relative solvent accessibility (RSA) of peptide residues mapping in HA and M2 protein was calculated using NACCESS (48) and average solvent accessibility (ASA) of peptides was computed upon them as reported elsewhere (42, 49). Venn diagrams were generated using the nVennR package version 0.2.3 (50).

## 3 Results and discussion

### 3.1 Shared peptidome space between selected bacteria and respiratory viruses

Cross-reactive immunity is much more likely to happen between antigens with high sequence similarity. However, because B and T cells recognize small regions in protein antigens (epitopes), cross-reactivity is better predicted by the volume of peptides (peptidome) that are shared between antigens (34). Thereby, we determined the shared peptidomes between 8 common respiratory viruses (IAV, IBV, HRV A, B and C, RSV A and B, and SARS-CoV-2) and 7 bacteria species, including BCG and those in MV130. To that end, we obtained the relevant proteomes and through a BLAST based approach (details in Methods) identified all unique peptides with identity ≥ 80% and length ≥ 8 in common between viruses and bacteria. The results of this analysis are summarized in **Table 2**. As noted by previous works, the volume of shared peptides increases with the size of the proteomes (34, 51). Thus, of all the studied bacteria and viruses, *K. pneumoniae* (KPN) followed by Bacille Calmette-Guérin (BCG) share with SARS-CoV-2 (SARS) the largest number of peptides, 138 and 102, respectively.

**Table 2:** Size of the shared peptidome between bacteria in MV130 and respiratory viruses.

	ORF	IAV	IBV	HRVA	HRVB	HRVC	RSVA	RSVB	SARS
*Streptococcus pneumoniae* (SPN)	1861	12	17	8	13	17	25	31	52
*Staphylococcus aureus* (SAU)	2767	23	36	8	12	10	39	46	68
*Staphylococcus epidermidis* (SEP)	2282	27	30	11	14	10	27	30	53
*Klebsiella pneumoniae* (KPN)	5770	48	57	19	32	21	53	53	138
*Branhamella catarrhalis* (BCA)	1607	15	18	11	10	9	27	20	38
*Haemophilus influenzae* (HIN)	1597	25	18	4	9	8	31	20	50
Bacille Calmette-Guérin (BCG)	4045	46	41	13	27	25	32	32	102
MV130	15884	139	163	54	79	72	185	183	360

ORF, Open Reading Frame; IAV, Influenza A virus; IBV, Influenza B virus; HRVA, human rhinovirus A; HRVB, human rhinovirus B; HRVC, human rhinovirus C, RSVA, Respiratory Syncytial virus A, RSVB, Respiratory Syncytial virus B; SARS, SARS-CoV-2. Whole dataset available in **Supplementary Dataset 1**.

**Figure 1:**
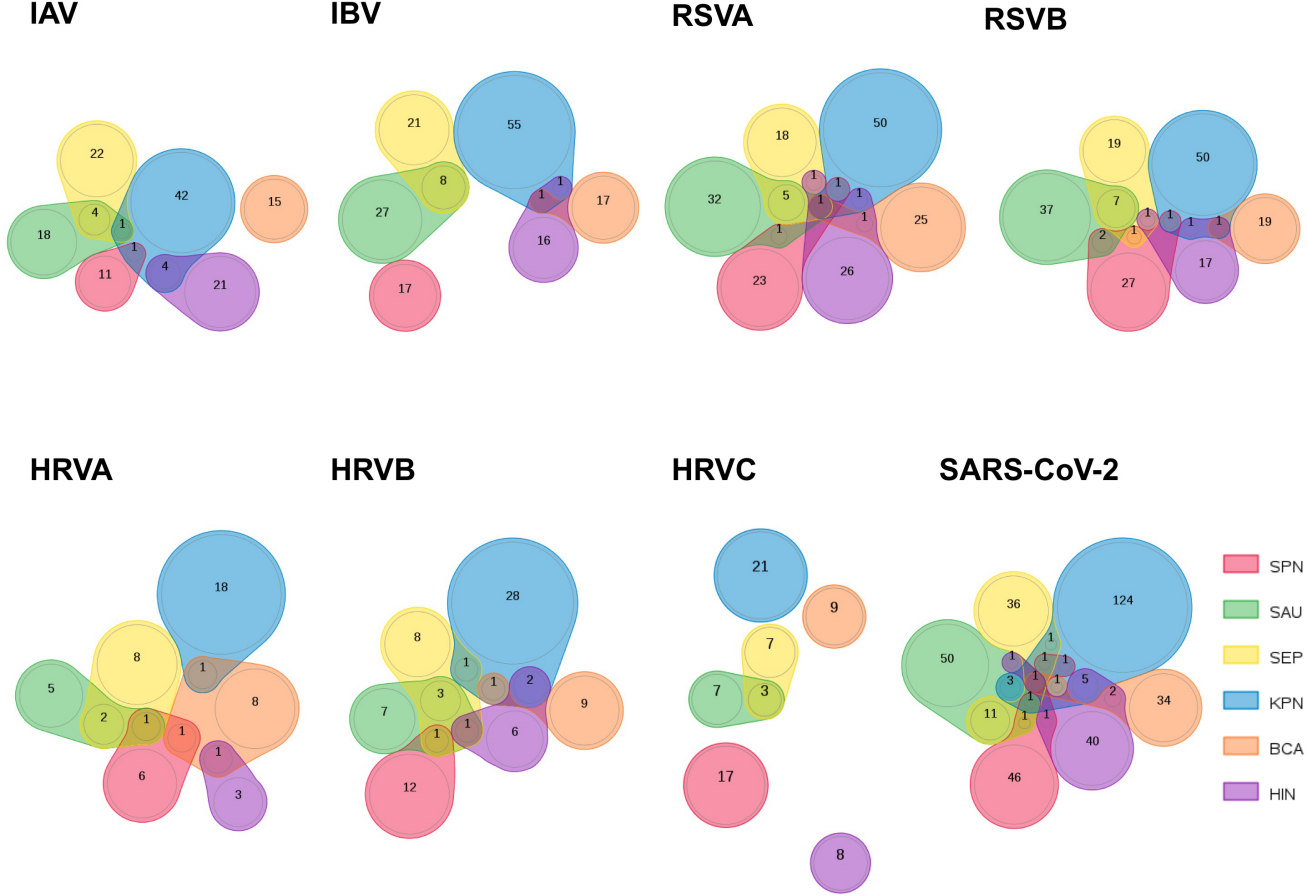
Comparison of peptidomes shared by respiratory viruses and bacteria in MV130. The sets of peptides that are shared between 8 respiratory viruses and each bacterium included in the MV130 formulation were compared and represented using Venn diagrams to visualize the overlaps. The number of peptides in overlapping and non-overlapping regions is indicated. The represented viruses are (from left to right and up to down): IAV: Influenza A virus; IBV: Influenza B virus; HRVA: human rhinovirus A; HRVB: human rhinovirus B; HRVC: human rhinovirus C, RSVA: Respiratory Syncytial virus A, RSVB: Respiratory Syncytial virus B; SARS: SARS-CoV-2. The six bacteria species included in MV130 are indicated and colored as follows: *S. pneumoniae* (SPN, red); *S. aureus* (SAU, green); *S. epidermidis* (SEP, yellow); *K. pneumoniae* (KPN, blue); *B. catarrhalis* (BCA, orange); *H. influenzae* (HIN, purple).

Interestingly, the peptides that are shared between the selected respiratory viruses and each bacterium in MV130 are largely distinct, as highlighted by the Venn diagrams depicted in **Figure 1**. Within the set of peptides shared by any of the viruses and a particular MV130 bacterium, only a handful coincides with those shared with another bacterium. An exception occurs in the case of *S. aureus* (SAU) and *S. epidermidis* (SEP), whose shared peptidomes with viruses are highly overlapping, as one could expect for they are closely related bacteria. As a result, the number of peptides that are shared between MV130, as a whole, and the different viruses is close to the sum of peptides that are shared by each bacterium in MV130 (**Table 2**). This effect is far from trivial as indicates that combining different bacteria, as those in MV130, increases the chance for cross-reactive immunity. Moreover, it supports the noted view (34) that a diverse microbiota helps to fight viral infections (26) by enhancing cross-reactive immunity.

### 3.2 Potential cross-reactive immunity from MV130 to IAV

The large size of the peptidome shared between MV130 and the studied respiratory viruses is indicative that MV130 could be a major source of cross-reactive immunity against these viruses. However, for the realization of cross-reactive immunity these peptides must be recognized by the adaptive immune system. Here, we explored such realization for IAV, since it causes respiratory infections prevented by MV130 (10, 11) and MV130 confers resistance to lethal IAV challenges in mice (12). To that end, we predicted the potential of the peptides shared between MV130 and IAV to represent B and T cell epitopes. Briefly, peptides predicted to bind class I and/or class II MHC molecules (in human HLA molecules) were considered CD8 and CD4 T cell epitopes, respectively (details in Methods). Moreover, for fulfillment of T cell cross-reactivity we required that both, the IAV peptide and the equivalent MV130 peptide, bind to the same MHC/HLA molecule. Because B cell reactivity is somewhat less predictable than T cell reactivity, B cell cross-reactivity was considered when either the IAV peptide or the equivalent MV130 peptide had a B cell epitope score ≥ 0.4 (details in Methods). In **Table 3**, we summarize the results of this analysis. Many more cross-reactive CD8 than CD4 T cell epitopes were predicted. This is the expected result, as CD4 T cell epitopes are longer than CD8 T cell epitopes and there are few peptides in the shared peptidome with the size required to be CD4 T cell epitopes. Nonetheless, we realize that we identified fewer cross-reactive T cell epitopes than in a previous work (33, 34) using Immune Epitope Database (IEDB) MHC-binding models through the RESTful interface (52).

**Table 3:** Potential cross-reactive epitopes between MV130 and IAV.

	IAV ^(1)^	B ^(2)^	CD8 T ^(H)^	CD4 T ^(H)^	CD8 T ^(M)^	CD4 T ^(M)^
*Streptococcus pneumoniae* (SPN)	12	2	4	1	1	0
*Staphylococcus aureus* (SAU)	25	5	2	0	3	0
*Staphylococcus epidermidis* (SEP)	27	6	7	1	7	1
*Klebsiella pneumoniae* (KPN)	48	10	13	1	4	1
*Branhamella catarrhalis* (BCA)	15	7	4	1	1	0
*Haemophilus influenzae* (HIN)	25	4	7	1	5	1
MV130	139	34	37	5	21	3

^1^Number of shared peptides between IAV (Puerto Rico 8 Strain) and bacteria, ^2^ number of cross-reactive B cell epitopes, ^H^ number of T cell epitopes restricted by human MHC molecules; ^M^ number of T cell epitopes restricted by mouse MHC molecules.

As expected, the number of distinct cross-reactive B and T cell epitope peptides correlated with the number of shared peptides and hence with the size of the proteomes of the bacteria in MV130 (**Table 2** and **Supplementary Dataset 1**). These cross-reactive peptide epitopes were private, differ between bacteria, and so the potential cross-reactive immunity of the MV130 formulation is the sum of each individual bacterium. Hence, MV130 appears to be a truly enhanced source of cross-reactive immunity. We next examined in detail the predicted cross-reactive epitopes to evaluate to what extent they could provide protective immunity against IAV.

### 3.3 Cross-reactive T cell epitopes between MV130 and IAV target numerous HLA molecules

MHC molecules restricting T cells are highly polymorphic in humans (HLA), bind/present distinct sets of peptides that can nonetheless be overlapping, and are expressed at different frequency in the population depending on ethnicity and geography (53, 54). Subsequently, the immunogenicity of any given T cell epitope varies between individuals, as it is contingent on the expression/presence of the specific MHC molecule restricting the epitopes. In **Table 4**, we show all the potential cross-reactive T cell peptide epitopes along with their predicted HLA binding/presentation profiles (details in Methods). We also show the MHC molecules expressed by C57BL/6 and BALB/c mice strains that can present these same peptide epitopes (peptides that were only predicted to bind to mouse MHC molecules are not shown). Given the key role of CD8 T cells in clearing and containing viral infections we will pay particular attention to cross-reactive CD8 T cell epitopes.

**Table 4:** Potential cross-reactive T cell epitopes between MV130 and IAV.

IAV ^(1)^ ACN|ANTIGEN	MV130 ^(2)^ ACN|BACTERIA	IAV SEQ ^(3)^	HIT SEQ ^(4)^	ID (%) ^(5)^	HLA I ^(6)^	HLA II ^(7)^	H-2I ^(8)^	H-2II ^(9)^	IEDB ^(10)^
YP_418248**|** PB1-F2	WP_003657597.1|BCA|	GQQTPKLEHRN	GQLTGKLEHRN	81.81	HLA-A*31:01, HLA-A*33:01, HLA-B*15:01	–	–	–	
NP_040983**|** NS2	WP_041786745.1|BCA|	WLIEEVRHKLK*	WLIELLRHKLK*	81.81	HLA-A*02:01, HLA-A*02:03, HLA-A*02:06, HLA-A*03:01, HLA-A*11:01, HLA-B*07:02, HLA-B*08:01, HLA-B*40:01, HLA-B*44:02, HLA-B*44:03	–	H-2-Kk, H-2-Kq	–	148643
NP_040982**|** NP	WP_164927877.1|HIN|	SGYDFEREGY*	KGYQFEREGY*	80	HLA-A*30:02	–	–	–	21577
NP_040985**|** PB1	WP_001831697.1|SEP|	LNPFVSHKEI*	LNGFVPHKEI*	80	HLA-B*51:01	–	–	H-2-IEd	–
NP_040978**|** M1	YP_005224566.1|KPN|	PLKAEIAQRL*	PTRAEIAQRL*	80	HLA-A*31:01, HLA-A*33:01	–	H-2-Kk	–	48376
NP_040981**|** NA	YP_005229006.1|KPN|	TFFLTQGALL*	TFFLTFGSLL*	80	HLA-A*23:01, HLA-A*24:02, HLA-B*08:01	–	H-2-Kb, H-2-Kd	–	127810
NP_040987**|** PB2	YP_005226190.1|KPN|	LRISSSFSFG	LRIISSFGFG	80	HLA-A*32:01	–	–	–	2133253
NP_040985**|** PB1	WP_003658761.1|BCA|	EKIRPLLIEG	EKIRFLLLEG	80	HLA-A*30:01	–	–	–	212044
NP_040985**|** PB1	WP_002484992.1|SEP|	MDVNPTLLFL*	MDVMPTLLHL*	80	HLA-A*02:06, HLA-A*26:01, HLA-A*68:02, HLA-B*35:01, HLA-B*51:01, HLA-B*53:01	–	H-2-Db, H-2-Dd, H-2-Dq, H-2-Kb, H-2-Kk, H-2-Kq, H-2-Lq	–	41282
NP_040978**|** M1	WP_002440602.1|SEP|	DKAVKLYRK*	DKLVKHYRKL*	80	HLA-A*30:01, HLA-A*32:01, HLA-A*33:01, HLA-B*08:01	HLA-DRB1*13:02	H-2-Db, H-2-Dd, H-2-Kb	–	231836
NP_040984**|** NS1	YP_005225903.1|KPN|	LGDAPFLDRL*	LGIAPLLDRL*	80	HLA-B*51:01	–	H-2-Dd, H-2-Kb	–	_
NP_040983**|** NS2	YP_005226633.1|KPN|	RDSLGEAVMR*	RDSLLEAVLR*	80	HLA-A*31:01, HLA-A*33:01, HLA-A*68:01, HLA-B*40:01	–	–	–	1846494
NP_040981**|** NA	WP_010869183.1|HIN|	SVRQDVVAMT	SVAQDVDAMT	80	HLA-A*02:06, HLA-A*26:01, HLA-A*68:02, HLA-B*35:01	–	H-2-Db	–	–
NP_040983 NS2	YP_005226684.1|KPN|	EIRWLIEEVR	EIRWMIEELR	80	HLA-A*26:01, HLA-A*33:01, HLA-A*68:01, HLA-A*68:02	–	–	–	–
NP_040987**|** PB2	WP_013107773.1|BCA|	QSLIIAARNI*	QSLIGAVRNI*	80	HLA-A*02:03	HLA-DRB1*11:01	–	–	128453
NP_040987**|** PB2	YP_005225299.1|KPN|	LRVRDQRGNV*	VRVRLQRGNV*	80	HLA-A*30:01, HLA-B*07:02	–	–	–	129735
NP_040980**|** HA	WP_005693451.1|HIN|	QNAINGITNK*	QNAIAGLTNK*	80	HLA-A*03:01, HLA-A*11:01, HLA-A*68:01	HLA-DRB1*15:01	–	H-2-IAs	128451
NP_040980**|** HA	YP_005226466.1|KPN|	LGAINSSLPF*	LGVINSGLPF*	80	HLA-A*32:01, HLA-B*15:01, HLA-B*35:01	–	–	–	–
NP_040978**|** M1	WP_010869065.1|HIN|	LTEVETYVLS	LLEVETPVLS	80	HLA-B*40:01	–	H-2-Kk, H-2-Kq	–	128060
NP_040985**|** PB1	YP_005228858.1|KPN|	KLRTQIPAE	KLREQIPAE	88.89	HLA-A*30:01	HLA-DPA1*02:01/DPB1*14:01	–	–	–
NP_040980**|** HA	WP_000260666.1|SPN|	TVLEKNVTV*	AVLEKNVTV*	88.9	HLA-A*02:01, HLA-A*02:03, HLA-A*02:06, HLA-A*32:01, HLA-A*68:02, HLA-B*08:01	HLA-DRB3*02:02	H-2-Db, H-2-Kb	–	238314
NP_040982**|** NP	WP_164927877.1|HIN|	GYDFEREGY*	GYQFEREGY*	88.9	HLA-A*30:02	–	–	–	128838
NP_040987**|** PB2	WP_001830401.1|SEP|	FVNRANQRL*	FVNRKNQRL*	88.9	HLA-A*02:03, HLA-A*02:06, HLA-A*33:01, HLA-A*68:02	–	H-2-Kb	–	97519
NP_040987**|** PB2	YP_005227236.1|KPN|	QSLIIAAR	QSLIIAAR	100	HLA-A*33:01	–	–	–	128453
NP_040980**|** HA	WP_000260666.1|SPN|	VLEKNVTV*	VLEKNVTV*	100	HLA-A*02:01, HLA-A*02:03	–	–	–	69459
NP_040983**|** NS2	WP_002468856.1|SEP|	FEEIRWLI*	FESIRWLI*	87.5	HLA-B*40:01	–	H-2-Kk, H-2-Kq	–	–
NP_040985**|** PB1	WP_005688698.1|HIN|	ALANTIEV*	ALANTIVV*	87.5	HLA-A*02:01, HLA-A*02:03	–	–	–	62904
NP_040985**|** PB1	WP_005693559.1|HIN|	RSKAGLLV*	RSKKGLLV*	87.5	HLA-A*30:01	–	–	–	–
NP_040985**|** PB1	YP_499926.1|SAU|	SMKLRTQI*	SPKLRTQI*	87.5	HLA-B*08:01	–	–	–	128581
NP_040987**|** PB2	WP_001832661.1|SEP|	PNEVGARI*	PNEVGRRI*	87.5	HLA-B*51:01	–	–	–	68545
NP_040984**|** NS1	YP_005228222.1|KPN|	ESDEALKM*	ESDELLKM*	87.5	HLA-A*01:01	–	–	–	97398
YP_006495785**|** PA-X	YP_005229545.1|KPN|	PREEKRQL*	PREEWRQL*	87.5	HLA-B*07:02	–	–	–	–
NP_040987**|** PB2	YP_500587.1|SAU|	FVNRANQR*	FVNRKNQR*	87.5	HLA-A*33:01	–	–	–	97519
NP_040978**|** M1	WP_000597995.1|SPN|	WLKTRPIL*	WLSTRPIL*	87.5	HLA-B*08:01	–	–	–	69642
NP_040981**|** NA	YP_005229237.1|KPN|	ITETIKSW*	IGETIKSW*	87.5	HLA-B*57:01, HLA-B*58:01	–	–	–	–
NP_040986**|** PA	WP_001830509.1|SEP|	VELAEKTM*	VELNEKTM*	87.5	HLA-B*40:01	–	H-2-Kk, H-2-Kq	–	–
NP_040983**|** NS2	WP_010976535.1|SPN|	LESSSEDL*	LESDSEDL*	87.5	HLA-B*40:01	–	–	–	–

^(1)^ Accession and antigen of IAV peptide, ^(2)^ Accession of bacteria peptide, ^(3)^ Sequence of IAV peptide, ^(4)^ Sequence of bacteria peptide, ^(5)^ Percentage of identity between IAV and bacteria peptides, ^(6)^ HLA I and ^(7)^ HLA II molecules, and ^(8)^ Mouse H2-I and ^(9)^ H2-II molecules predicted to bind both the IAV and bacteria peptides, ^(10)^ Accession of IAV T cell epitope in IEDB coinciding with IAV cross-reactive peptide (≥ 90% identity and ≥ 8 residues). * Peptides have a C-terminus compatible with cleavage by the proteasome.

**Figure 2:**
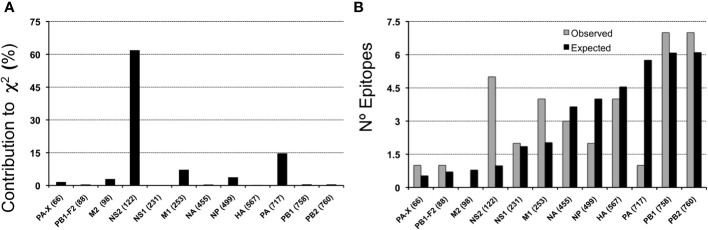
Antigen-size distribution of MV130 cross-reactive CD8 T cell epitopes in IAV. **(A)** Contribution to χ^2^ -statics of each IAV antigen for the distribution of cross-reactive epitopes according to the size of antigens **(B)** Representation of the number of observed (grey bars) and expected (black bars) cross-reactive CD8 T cell epitopes in each IAV antigen. The number of residues of each antigen is shown in parenthesis adjacent to the antigen name.

MV130 encompasses 37 unique peptide sequences consisting of cross-reactive CD8 T cell epitopes with IAV that are distributed through all IAV antigens but M2 (**Tables 3**, **4**). These epitopes are not distributed proportionally to the size of the IAV antigens as revealed by χ^2^ statistics (*p*< 0.005). The largest contributions to χ^2^ statistics are found in non-structural protein 2 (NS2), polymerase PA and M1 (**Figure 2A**). In particular, while NS2 and M1 bear more cross-reactive T cell epitopes than the expected, PA includes fewer than the expected (**Figure 2B**). The uneven distribution of cross-reactive CD8 T cell epitopes throughout the IAV proteome supports the specificity of T cell cross-reactivity. In fact, it is worth noting that 24 of the 37 cross-reactive peptides coincide with IAV-specific CD8 T cell epitopes deposited in the IEDB (**Table 4**).

The majority of potential MV130-IAV cross-reactive CD8 T cell epitopes can also be presented by more than one HLA I molecule and 5 of them can also be presented by HLA II molecules (**Table 4**). The combined phenotypic frequency in the population of all HLA I molecules restricting these cross-reactive CD8 T cell epitopes would imply that MV130 could elicit cross-reactive CD8 T cell immunity to IAV will in ≥ 95% of the population the regardless of their genetic background (details in Methods). Bacteria eliciting memory CD8 T cells capable of recognizing antigens displayed by virally infected cells can occur through antigen cross-presentation (55). This mechanism enables professional antigen presenting cells to redirect antigens taken from the extracellular milieu for presentation in the context of HLA I molecules and prime CD8 T cells against extracellular antigens (55). There are distinct cross-presentation pathways, including some that are proteasome dependent, just like the classical class I antigen presentation pathway (56). In this regard, 29 out of 37 shared peptides defining potential cross-reactive T cell epitopes have a C-terminus that is compatible with cleavage by the proteasome (**Table 4**, details in **Supplementary Dataset 1**). Vaccines consisting of inactivated virus or viral antigens can likely induce protective anti-viral CD8 T cell memory thanks to this same mechanism.

Since only 5 potential cross-reactive CD4 T cell epitopes were detected (**Tables 3**, **4**), it could be argued that MV130 may not induce enough T helper (Th) cells to support the whole antiviral potential of cross-reactive CD8 T cells. However, the percentage of the world population that could respond to any of these 5 cross-reactive CD4 T cell epitopes is actually about 33.7% as computed using the frequency of the relevant HLA II molecules (see Methods). Moreover, it is likely that there are many more cross-reactive CD4 T cell epitopes than those detected through our methodology. In fact, CD4 T cells are in general more cross-reactive than CD8 T cells and can recognize many distinct peptides despite sharing little sequence similarity (57, 58). In this context, our findings explain the increase (~ 30-fold) in the influenza virus-specific CD8 T cell response, following MV130 treatment in patients with RRTI (5).

In this study, we trusted the detected T cell cross-reactivity between MV130 and IAV on the predicted binding of shared peptides to the same HLA alleles. However, binding of peptides to MHC molecules is not enough to guarantee T cell reactivity *in vivo*. Thus, it has been shown that peptides with high binding affinity for MHC molecules *in vitro* can nonetheless be excluded from T cell recognition *in vivo*, very likely due to a lack of appropriate antigen processing (59). Hence, the actual realization of the predicted T cell cross-reactivity from MV130 to IAV is contingent on the appropriate processing of antigens. This processing will involve, on the one hand, the uptake of bacteria by antigen-presenting cells, processing of bacteria antigens and presentation of peptide antigens by HLA molecules to prime T cells. On the other hand, it will require that IAV infected cells and/or antigen-presenting cells that have captured IAV antigens do also process the antigens and present the counterpart peptides by the same HLA molecules. These antigen processing events were not taken in consideration in this study because of their complexity and because they are less predictable than binding to MHC molecules. Thereby, *in vivo* studies are required to confirm the predicted T cell cross-reactivity of MV130 to IAV. Given that there are cross-reactive CD8 T cell epitopes restricted by both human and mouse MHC molecules (**Table 4**), mouse infectious disease models could be used to identify cross-reactive immunity relevant to humans and contribution to IAV protection.

### 3.4 Cross-reactive B cell epitopes between MV130 and IAV could be neutralizing

Preexisting protective cross-reactive immunity to virus is more often linked to T cells (21, 60). However, cross-reactive antibodies between viruses and bacteria have been reported (61, 62) and we have previously shown that the spike protein of SARS-CoV-2 includes potentially neutralizing B cell epitopes that are shared with bacteria targeted by diphtheria-tetanus vaccines (33, 34). Thereby, we investigated MV130 cross-reactive B cell epitopes mapping on the ectodomains of IAV proteins that are known be targeted by antibodies hampering viral entry. These proteins are HA, NA and M2. We found 8 of such cross-reactive B cell epitopes in HA, one in M2 and none in NA (**Table 5**). The average solvent accessibility (ASA) of these cross-reactive B cell epitopes (details in Methods) is greater than 25% (**Table 4**), indicating that they are readily accessible to antibodies. Moreover, we verified that 5 of these cross-reactive B cell epitopes coincide with experimentally determined B cell epitopes deposited at IEDB, including LLTEVETP, which matches a known B cell epitope in M2 annotated as neutralizing in IEDB (targeted by neutralizing antibodies). M2 is a proton-selective transmembrane ion channel located in the viral envelope required for the efficient release of the IAV genome into host cells (63, 64). Interestingly, only the N-terminal region of M2 (residues 1-22) surfaces the virion membrane and it is in this precise region that lays LLTEVETP (residues 3-10). This region is extremely conserved across all reported influenza A viruses and hence cognate antibodies could provide heterotypic influenza immunity. Although our approach did not yield any cross-reactive T cell epitope in M2, CD4 and CD8 T cell epitopes in M2 ectodomain have been reported that can mediate protective immunity to IAV (65, 66). Whether these T cell epitopes could have been predicted as cross-reactive using less stringent criteria of similarity is something to consider. The main target of neutralizing antibodies against IAV is however HA, as this protein dominates the surface of IAV and facilitates viral entry into host cells (57). Hence, we investigated the neutralizing potential of HA cross-reactive B cell epitopes by mapping them on the 3D-structure of HA and examining relevant structural information.

**Table 5:** Potential MV130-IAV cross-reactive B cell epitopes on ectodomains of HA and M2.

IAV ^(1)^ ACN/ANTIGEN	MV130 ^(2)^ ACN|BACTERIA|	ID ^(3)^ (%)	IAV PEP	MV130 PEP	B ^(4)^	ASA ^(5)^ (%)	IEDB (6)
NP_040980 HA	WP_005693451.1|HIN|	80.0	QNAINGITNK	QNAIAGLTNK	0.4	44.50	1180011
NP_040980 HA	YP_005224881.1|KPN|	87.5	AIAGFIEG	AIAGQIEG	0.6	27.43	163243*
NP_040980 HA	YP_005229187.1|KPN|	87.5	LSRGFGSG	LSRGFASG	0.4	25.80	538658
NP_040980 HA	WP_161375000.1|SEP|	87.5	GIITSNAS	GAITSNAS	0.7	31.03	–
NP_040980 HA	YP_005220833.1|KPN|	87.5	LCRLKGIA	LCRLFGIA	0.5	30.05	–
NP_040980 HA	WP_003659222.1|BCA|	87.5	REKVDGVK	RQKVDGVK	0.7	50.02	–
NP_040980 HA	YP_005227766.1|KPN|	87.5	PKESSWPN	PDESSWPN	2.0	59.89	–
NP_040980 HA	WP_002440219.1|SEP|	87.5	KKGKEVLV	KKGKVVLV	0.4	32.76	151030
NP_040979 M2	YP_499789.1|SAU|	87.5	LLTEVETP	LLTMVETP	0.4	82.20	59316*

**^(1)^** Accession and antigen source of IAV peptide, ^(2)^ Accession of bacteria antigen from BLAST hit, ^(3)^ Percentage of identity between MV130 peptide hit to equivalent IAV peptide, **^(4)^** B cell reactivity as predicted by Bepipred1.0, **^(^**^5^**^)^** Average solvent accessibility of IAV peptides computed after RSA values of peptides residues obtained from the relevant 3D-structures (HA: PDB 1RU7; & M2: PDB 5DLM). ^(5)^ Accession of B cell epitope in IEDB coinciding with cross-reactive epitope (≥ 90% identity and ≥ 8 residues). * Epitopes annotated in IEDB as neutralizing (targeted by neutralizing antibodies).

**Figure 3:**
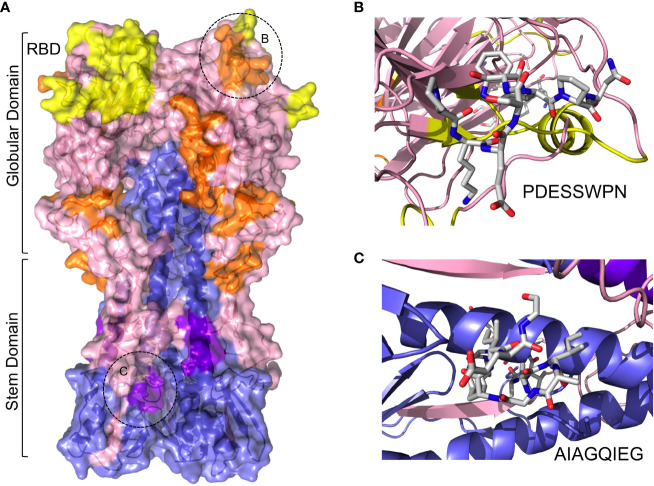
Cross-reactive B cell epitopes between MV130 and HA. **(A)** Molecular surface of HA with ribbon structure underneath showing cross-reactive B cell epitopes. HA1 and HA2 chains have been colored in blue and pink, respectively, and the RBD in yellow. Cross-reactive epitopes mapping in HA1 and HA2 are colored in orange and deep purple, respectively. Globular and stem domains are labeled as well as the RBD. The regions circled and labeled as B and C points to cross-reactive B cell epitopes PDESSWPN and AIAGQIEG, which are zoomed in the corresponding right panels. **(B)** Stick rendering of cross-reactive B cell epitope PDESSWPN. **(C)** Detail of cross-reactive B cell epitope AIAGQIEG in stick rendering.

The mature HA includes 3 HA1 and 3 HA2 subunits –derived from the same polypeptide chain – that fold into a trimeric structure depicting a globular head domain and a stem domain (**Figure 3A**) The globular domain is made of HA1 subunits and includes the receptor binding domain (RBD), which attach sialic acid in various membrane proteins, facilitating viral entry (67). This globular domain, particularly the vicinity of the RBD, is hence the subject of recognition by many neutralizing antibodies (68). Most of the selected cross-reactive B cell epitopes map in the surface of the globular domain, relatively close to the RBD (**Figure 3A**). Thus, one could speculate that antibodies recognizing these B cell epitopes could impede IAV attachment to host cells and block viral entry. This effect can be readily visualized for the cross-reactive B cell epitope PKESSWPN (HA residues 120-127) (**Figures 3A, B**), since this epitope is adjacent to the 130-loop (residues number 134-142) which takes part of RBD (67). To a lesser extent, the stem domain can also be the target of neutralizing antibodies, which generally interfere with conformational changes required for IAV membrane fusion (67, 68). Interestingly, one of the cross-reactive B cell epitopes in the stem domain of HA is AIAGFIEG (**Figures 3A, C**), which coincides with a known B cell epitope recognized by neutralizing antibodies (**Table 5**) (69). Thereby, we can foresee that the neighboring cross-reactive B cell epitope QNAINGITNK could also be neutralizing (**Table 5** and **Figure 3A**).

## 4 Conclusion and limitations

Our findings indicate that MV130 is an enhanced source of cross-reactive immunity to common respiratory viruses and in particular to IAV, which result of combining distinct bacteria in the same formulation. MV130 indeed present many potential cross-reactive T cell epitopes with IAV that are restricted by a broad spectrum of HLA molecules. Hence, MV130 could induce anti-IAV T cell responses in individual regardless of their genetic background. Likewise, MV130 encompasses many potential cross-reactive B cell epitopes mapping in critical regions of IAV membrane proteins, so that neutralizing antibodies may also be induced. In sum, cross-reactive adaptive immunity surely contributes, together with trained innate immunity, to the heterologous antiviral immunity associated with MV130.

It is worth noting some limitations that could affect our results. First, we relied heavily on sequence similarity to anticipate potential cross-reactive epitopes. However, antigen receptors can recognize diverse antigens and the structural bases for their promiscuity are ill defined. Antigen recognition by T cell receptors can be particularly subtle (70). Thus, while an individual T cell clone can cross-reactively recognize many diverse peptides (71), a single amino acid change in a TCR contact of a cognate peptide can greatly alter T cell recognition (72). Secondly, we did not take in consideration antigen processing to predict potential cross-reactive T cell epitopes, nor evaluated the solvent accessibility of linear B cell epitopes in bacteria. These considerations along with the fact that epitope prediction is not a precise science could limit the realization of the predicted cross-reactivity between MV130 and IAV. Therefore, it is important to stress the need for experimental validation of the cross-reactive epitopes predicted in this work.

## Data availability statement

The original contributions presented in the study are included in the article/**Supplementary Material**. Further inquiries can be directed to the corresponding authors.

## Author contributions

Conceptualization: PAR & JLS. Methodology: EML, AR-C & PAR. Data Analysis: AB-P, EML & HFP-P. Investigation: AB-P, EML, PAR & JLS. Writing-Original Draft: AB-P, HFP-P & PAR. Final Writing & Editing: PAR & JLS.

## Supplementary material

Supplementary Data Sheet 1 Excel file with the amino acid sequences of shared peptides between MV130 and IAV and predicted cross-reactivity.
