# Corrigendum: Combining different bacteria in vaccine formulations enhances the chance for antiviral cross-reactive immunity: a detailed *in silico* analysis for influenza A virus

**DOI:** 10.3389/fimmu.2023.1284628

**Published:** 2023-09-08

**Authors:** Andrés Bodas-Pinedo, Esther M. Lafuente, Hector F. Pelaez-Prestel, Alvaro Ras-Carmona, Jose L. Subiza, Pedro A. Reche

**Affiliations:** ^1^Children’s Digestive Unit, Institute for Children and Adolescents, Hospital Clinico San Carlos, Madrid, Spain; ^2^Department of Immunology & O2, Faculty of Medicine, University Complutense of Madrid, Ciudad Universitaria, Pza. Ramón y Cajal, Madrid, Spain; ^3^Inmunotek, Alcalá de Henares, Spain

**Keywords:** MV130, bacteria, respiratory viruses, cross-reactivity, epitope, influenza A virus

In the published article, there was an error in **Table 1** as published. In row 1, column 2, ¨Accesion” was misspelled. It should be “Accession”. In row 13, column 1, “Klebisella” was misspelled. It should be “Klebsiella”. In addition, in row 9 for SARS-CoV2, column 2 was incorrect (the accession number listed was GCF_000009445, but should have been NC_045512) and column 2 was empty but should have been 12. The corrected **Table 1** and its caption appear below.

**Table 1:** Amino acid sequences from pathogens and vaccines considered in this study.

Pathogen	NCBI Accession	Proteins/CDS
Influenza A virus (IAV)	GCF_000865725	12
Influenza B virus (IBV)	GCF_000820495	10
Human rhinovirus A (HRVA)	NC_038311	1
Human rhinovirus B (HRVB)	NC_038312	1
Human rhinovirus C (HRVC)	NC_009996	1
Respiratory syncytial virus A (RSVA)	NC_038235	11
Respiratory syncytial virus A (RSVB)	NC_001781	11
Severe acute respiratory syndrome coronavirus 2 (SARS-CoV-2)	NC_045512	12
Bacille Calmette-Guérin (BCG)	GCF_000009445	4034
*Branhamella catarrhalis* (BCA)	GCF_000092265	1607
*Haemophilus influenzae* (HIN)	GCF_000027305	1597
*Klebsiella pneumoniae* (KPN)	GCF_000240185	5779
*Staphylococcus aureus* (SAU)	GCF_000013425	2767
*Staphylococcus epidermidis* (SEP)	GCF_000007645	2282
*Streptococcus pneumoniae* (SPN)	GCF_000007045	1861
MV130*		15893

* MV130 includes all bacteria but BCG.

In the published article, there was an error in **Table 2** as published. “Klebisella” was misspelled. It should be “Klebsiella”. The corrected **Table 2** and its caption appear below.

**Table 2:** Size of the shared peptidome between bacteria in MV130 and respiratory viruses.

	ORF	IAV	IBV	HRVA	HRVB	HRVC	RSVA	RSVB	SARS
*Streptococcus pneumoniae* (SPN)	1861	12	17	8	13	17	25	31	52
*Staphylococcus aureus* (SAU)	2767	23	36	8	12	10	39	46	68
*Staphylococcus epidermidis* (SEP)	2282	27	30	11	14	10	27	30	53
*Klebsiella pneumoniae* (KPN)	5770	48	57	19	32	21	53	53	138
*Branhamella catarrhalis* (BCA)	1607	15	18	11	10	9	27	20	38
*Haemophilus influenzae* (HIN)	1597	25	18	4	9	8	31	20	50
Bacille Calmette-Guérin (BCG)	4045	46	41	13	27	25	32	32	102
MV130	15884	139	163	54	79	72	185	183	360

ORF, Open Reading Frame; IAV, Influenza A virus; IBV, Influenza B virus; HRVA, human rhinovirus A; HRVB, human rhinovirus B; HRVC, human rhinovirus C; RSVA, Respiratory Syncytial virus A, RSVB: Respiratory Syncytial virus B; SARS, SARS-CoV-2. Whole dataset available in **Supplementary Dataset 1**.

In the published article, there was an error in **Table 3** as published. “Klebisella” was misspelled. It should be “Klebsiella”. In addition, the scientific names of bacteria were not in italic. The corrected **Table 3** and its caption appear below.

**Table 3:** Potential cross-reactive epitopes between MV130 and IAV.

	IAV (1)	B ^(2)^	CD8 T ^(H)^	CD4 T ^(H)^	CD8 T ^(M)^	CD4 T ^(M)^
*Streptococcus pneumoniae* (SPN)	12	2	4	1	1	0
*Staphylococcus aureus* (SAU)	25	5	2	0	3	0
*Staphylococcus epidermidis* (SEP)	27	6	7	1	7	1
*Klebsiella pneumoniae* (KPN)	48	10	13	1	4	1
*Branhamella catarrhalis* (BCA)	15	7	4	1	1	0
*Haemophilus influenzae* (HIN)	25	4	7	1	5	1
MV130	139	34	37	5	21	3

^1^ Number of shared peptides between IAV (Puerto Rico 8 Strain) and bacteria, ^2^ number of cross-reactive b cell epitopes, ^H^ number of T cell epitopes restricted by human MHC molecules, ^M^ number of T cell epitopes restricted by mouse MHC molecules.

In the published article, there was an error in the legend for **Figure 1** as published. HRVA and HRVB, standing for human rhinovirus A and B, respectively, missed the relevant “A” and “B”. The corrected legend appears below.

**Figure 1:**
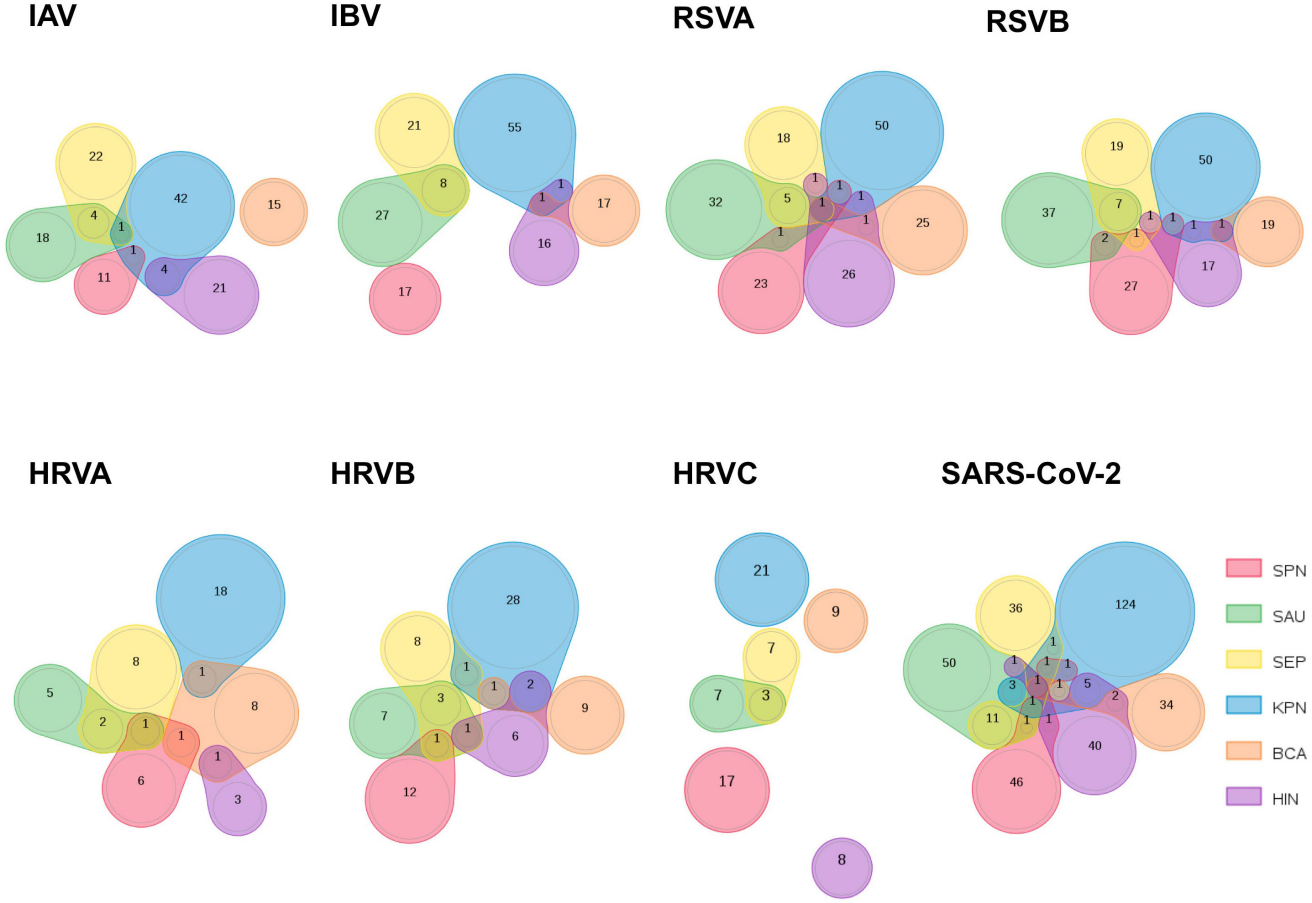
Comparison of peptidomes shared by respiratory viruses and bacteria in MV130. The sets of peptides that are shared between 8 respiratory viruses and each bacterium included in the MV130 formulation were compared and represented using Venn diagrams to visualize the overlaps. The number of peptides in overlapping and non-overlapping regions is indicated. The represented viruses are (from left to right and up to down): IAV: Influenza A virus; IBV: Influenza B virus; HRVA: human rhinovirus A; HRVB: human rhinovirus B; HRVC: human rhinovirus C; RSVA: Respiratory Syncytial virus A; RSVB: Respiratory Syncytial virus B; SARS: SARS-CoV-2. The six bacteria species included in MV130 are indicated and colored as follows: *S. pneumoniae* (SPN, red); *S. aureus* (SAU, green); *S. epidermidis* (SEP, yellow); *K. pneumoniae* (KPN, blue); *B. catarrhalis* (BCA, orange); *H. influenzae* (HIN, purple).

In the published article, there was an error in the **Abstract**. “Klebisella” was misspelled. It should be “Klebsiella”

A correction has been made to the **Abstract**. The corrected sentence appears below.

“The bacteria selected in this work were Bacillus Calmette Guerin and those included in the poly-bacterial preparation MV130: *Streptococcus pneumoniae*, *Staphylococcus aureus*, *Staphylococcus epidermidis*, *Klebsiella pneumoniae*, *Branhamella catarrhalis* and *Haemophilus influenzae*.”

In the published article, there was an error in the **Methods** section. HLA, standing for human leukocyte antigen, was used twice in Methods instead of MHC (major histocompatibility complex). MHCs are known as HLAs in humans, as indicated later in the Results section.

A correction has been made to the **Methods** section, subsection *2.3 Prediction of T and B cell epitopes*. This sentence previously stated:

“Binding of a peptide to a given HLA I molecule was considered to occur at a 2% Rank cutoff given by both RANKPEP and NetMHCpan, which allows selecting weak and strong binders.”

The corrected sentence appears below.

“Binding of a peptide to a given MHC I molecule was considered to occur at a 2% Rank cutoff given by both RANKPEP and NetMHCpan, which allows selecting weak and strong binders.”

Likewise, a correction has also been made to the **Methods** section, subsection *2.5 Other procedures*. This sentence previously stated:

“The percentage of the world population that could respond to CD8 and CD4 T cell epitopes (population coverage) was computed after their HLA binding profiles using a command line version of EPISOPT (44) and the IEDB PPC tool at http://tools.iedb.org/tools/population/iedb_input (45), respectively, considering HLA allele expression for the entire world population.”

The corrected sentence appears below.

“The percentage of the world population that could respond to CD8 and CD4 T cell epitopes (population coverage) was computed after their MHC binding profiles using a command line version of EPISOPT (44) and the IEDB PPC tool at http://tools.iedb.org/tools/population/iedb_input (45), respectively, considering the relevant allele expression for the entire world population.”

The authors apologize for these errors and state that this does not change the scientific conclusions of the article in any way. The original article has been updated.

